# PP39 Co-Creation Of A European Digital Health Technology Assessment Framework: The EDiHTA EU-Funded Project

**DOI:** 10.1017/S0266462324002113

**Published:** 2025-01-07

**Authors:** Emmanouil Tsiasiotis, Rossella Di Bidino, Dario Sacchini, Americo Cicchetti

## Abstract

**Introduction:**

The widespread adoption of digital health technologies (DHTs) brings new methodological challenges to health technology assessment (HTA). Different tools, guidelines, and methods have been defined by different stakeholders at national/international levels. In this scenario, the real thread for HTA is the lack of coordination and harmonization in the efforts to finalize the definition of an HTA framework for DHTs.

**Methods:**

The European Digital Health Technology Assessment (EDiHTA) project aims to provide the first framework co-created by all stakeholders along the value chain for DHTs. The consortium is comprised of 15 partners from nine countries (Italy, the Netherlands, Denmark, Spain, Germany, Norway, UK, Belgium, and Poland). Stakeholders included are HTA agencies, regulators, hospitals, patient/citizen representatives, academics/universities, notified bodies, and developers. The project covers all types of DHTs and Technology Readiness Levels (TRLs). A multistakeholder, multidomain, and modular approach is adopted with the final goal to provide a validated, ready-to-use digital HTA framework for DHTs for the European ecosystem.

**Results:**

The EDiHTA is articulated in eight working packages and covers four years. Starting from the mapping of the current scenario, after the definition of a common taxonomy, EDiHTA will develop a DHT-dedicated HTA framework. A common digital platform to support the assessment will be developed. Both the HTA framework and platform will be tested, refined, and validated through a piloting process involving disparate DHTs (mApp, telemedicine, artificial intelligence, and others) with different TRLs, available in different countries, with the participation of all stakeholders. As a consequence, evidence requirements will be defined and made more transparent; this will support health technology.

**Conclusions:**

The EDiHTA project will deliver the first European digital HTA framework able to support and inform decision-making (at the macro [policy], meso [management providers], and micro [clinicians] level) towards faster and safer access to DHTs of added value, taking European regulations and legal requirements into account and assessing all HTA domains.

